# 

**Published:** 1888-08

**Authors:** 


### LIST OF CONTRIBUTORS.

---

Angell, Dr. Edward B., Rochester, N. Y.
Baldy, Dr. J. M., Philadelphia, Pa.
Banta, Dr. Rollin Ledrue, Buffalo, N. Y.
Biggs, Dr. Hermann M., New York.
Billings, Dr. Frank S., Lincoln, Neb.
Campbell, Dr. F. R., Buffalo, N. Y.
Clark, Dr. Edward, Buffalo, N. Y.
Cohen, Dr. Bernard, Buffalo, N. Y.
Condict, Dr. Alice B., Hyderabad, India.
Crego, Dr. Floyd S., Buffalo, N. Y.
Cushing, Dr. Clinton, San Francisco, Cal.
Dagenais, Dr. A., Buffalo, N. Y.
Durant, Dr. C. Ferdinand, Lockport, N. Y.
Greene, Dr. W. D., Buffalo, N. Y.
Harrington, Dr. F. A., Buffalo, N. Y.
Hartwig, Dr. Marcell, Buffalo, N. Y.
Hubbell, Dr. Alvin A., Buffalo, N. Y.
Hurd, Dr. Arthur W., Buffalo, N. Y.
Ingraham, Dr. Henry D., Buffalo, N. Y.
Jacobson, Dr. Nathan, Syracuse, N. Y.
Krauss, Dr. Wm. C., Attica, N. Y.
Miller, Dr. John A., Buffalo, N. Y.
Moore, Dr. Edward M., Rochester, N. Y.
Mynter, Dr. Herman, Buffalo, N. Y.
Potter, Dr. Frank Hamilton, Buffalo, N. Y.
Potter, Dr. William Warren, Buffalo, N. Y.
Price, Dr. Joseph, Philadelphia, Pa.
Reed, Dr. Charles A. L., Cincinnati, Ohio.
Roberts, Dr. John B., Philadelphia, Pa.
Rohé, Dr. George H., Baltimore, Md.
Schultze, Dr. B. S., Jena, Germany.
Smith, Dr. Eugene A., Buffalo, N. Y.
Snow, Dr. Irving M., Buffalo, N. Y.
Stockton, Dr. Charles G., Buffalo, N. Y.
Tait, Mr. Lawson, Birmingham, England.
Tremaine, Dr. W. S., Buffalo, N. Y.
Wathen, Dr. Wm. H., Louisville, Ky.

### INDEX.

### A.

PAGE.
Abdominal Surgery, Some Recent Work in . . . . . . . . . . . . . 229
Abdominal Section, Complications following . . . . . . . . . . . . . 597
Abscess, A Contribution to the Study of Pelvic . . . . . . . . . . . . . . 369
Action of Calomel upon the Bile, etc. 33
Alexander (W.), A New Method of Treating Incontinence of Urine . . 34
Albuminuria in Bright's Disease . 488, 621
Allen Surgical Pump, A New Use of . 121
Alliance, A Physicians' Protective . . 267
American Resorts, etc. . . . . . . . . 702
American Medical Association, The Newport Meeting of . . . . . . . 760
Amputation of Thigh and Leg . . . . 457
Angell, Ed. B., M. D., Charcot's Method of Treating Ataxia, etc., . . 522
Antipyrin, Absorption and Excretion of . . . . . . . . . . . . . . . . 34
Antipyrin, Dangers of . . . . . . . 26
Antipyrin and Sweet Spirits of Nitre, 453
Antipyrin, Use of, with Quinine . . . 461
Antiseptics in Puerperal Disease, What is the Proper Use of . . . . . 121, 181
Annual of the Universal Sciences, . . 92
Applied Anatomy of the Nervous System . . . . . . . . . . . . . . . 48
Armamentarium Chirurgicum . . . . 699
Arthrectomia Talocruralis, . . . . . 541
Ataxia, Charcot's Method of Treating, Clinical Lectures on . . . . . 494, 621
Progressive Locomotor by Suspension . . . . . . . . . . . . . . 522
Ataxia, Locomotor, Treatment of, by Suspension . . . . . . . . . . . 669

### B.

Baldy, J. M., M. D., Complications following Abdominal Surgery . . . 597
Banta, R. L., M. D., What is the Proper Use of Antiseptics in Puerperal Disease? . . . . . . . . . . 181
Bantock, George Granville, M. D., F. R. C. S. Ed. Notes on Three Years' Ovariotomy Work in the Samaritan Free Hospital: Eighty-two cases without a death . . . . . 81
Bedside Record . . . . . . . . . . 95
Billings, Frank S., Vaccination . . . 581
Keratitis Contagiosa in Cattle . . 499
PAGE.
Billings, Frank S., M. D., Corn-stalk Disease in Cattle . . . . . . . . . 733
Blow on the Ear, with Peculiar Reflex Symptoms . . . . . . . . . . . . 202
Board of Health, New York, Reports of . . . . . . . . . . . . . . 214, 277
Bones, Lengthening of . . . . . . . 540
"Boo-Khar"—India Fever . . . . . 665
Biggs, Hermann M., M. D., The Principles of Treatment in Pulmonary Tuberculosis, etc. . . . . . . 633
Bright's Disease . . . . . . . . . . 493
Bladder, The Construction of a New . 460
Bronchitis, Varieties and Treatment of 356

### C.

Calculous Disease, etc. . . . . . . . 701
Calomel, Action of, upon the Bile, etc. 33
Campbell, F. R., A. M., M. D., Myths and Modern Therapeutics . . . . . 62
In Memoriam . . . . . . . . . 166
Successor to . . . . . . . . . 172
Cervical Lacerations, The Harmlessness of . . . . . . . . . . . . . . 165
Cerebellum, Tubercular Nodules of . . 439
Cesarean Section, The Abuse of . . . 262
Charcot's Method of Treating Progressive Locomotor Ataxia by Suspension, Translation . . . . . . . 522
Charter Revision Commission and the Buffalo Medical and Surgical Association . . . . . . . . . . . . . . 479
Chemist, An Expert . . . . . . . . 171
Chest in Health and Disease, Exploration of . . . . . . . . . . . . . 564
Chorea . . . . . . . . . . . . . . 187
Cholera, Duck . . . . . . . . . . 212
Chlormethyl . . . . . . . . . . . 542
Clark, Edward, M. D., Small-Pox Cases and their Management during the Recent Outbreak in Buffalo, etc. 97
Clavicles, A Subject without . . . . . 211
Clavicle, Treatment of, etc. . . . . . 363
Climatic Influence on the Morals . . 161
Clinical Lectures, Charcot . . . . . . 276
Close of Volume XXVIII., The . . . 759
Cocaine Poisoning, The Peculiarities of Chronic . . . . . . . . . . . . 527
Cocaine, What to Use . . . . . . . 88
Combination with the Medical Press of Western New York . . . . . . . 759
Combination of Medical Journals . . 752

PAGE.

Condict, Alice B., M. D., "Boo-Khar" in India Fever . . . . . . 665
Cohen, Bernard, M. D., Case of Traumatic Tetanus with Recovery . . . 199
Congenital Bony Occlusion of the Anterior Nares, Report of a Case of 74
Congress of American Physicians and Surgeons . . . . . . . 30, 37, 91, 169
Conium as a Local Anesthetic . . . . 34
Consideration of Some of the Recent Work on Abdominal Surgery . . . 229
Coroner's Jury Report . . . . . . . 164
Corn-stalk Disease in Cattle, The . . . 733
Correspondence. Ranney on Nervous Diseases . . . . . . . . . . . . 757
Coryza, Notes on the Treatment of Acute . . . . . . . . . . . . . . 302
Crego, Floyd S , M. D., Neurasthenia, or Nervous Exhaustion . . . . . . 295
Cremated, Eugenia to be . . . . . . 259
Creditors, Preferred . . . . . . . . 758
Croupous Inflammations, On the Nature of, with Special Reference to the Puerperium . . . . . . . . . 749
Croup, Tracheotomy in . . . . . . . 1
Crosson (D. M.) on Anomalous Ducts of Exits from the Parotid Glands in Front of the Ear . . . . . . . . . 162
Cushing, Clinton, M. D., A Contribution to the Study of Pelvic Abscess, 363

D.

Dagenais, A., M. D., Syphilis by Conception, Translation . . . . . . . 517
Dagenais, A., M. D., Alarming Hemorrhage from Rupture of the Hymen during First Coitus . . . . . . . . 250
Dangers of Antipyrin . . . . . . . 26
Davidson, Dr., Library of . . . . . 41, 89
Dictionary, Illustrated Encyclopedic Medical . . . . . . . . . . . . . 43
Diet Tables . . . . . . . . . . . . . 227
Diphtheria Treated by Chloral Hydrate, 213
Disinfection and Disinfectants . . . . 226
Durand, C. Ferdinand, M. B., Collapse from Rectal Injection . . . . . . . 456
Dystocia, due to Male Type of Pelvis and Hydrocephalus . . . . . . . 454

E.

Ear, Diseases and Injuries of . . . . 704
—and its Diseases . . . . . . . 275
Ear, A Blow on the, with Peculiar Reflex Symptoms . . . . . . . . 202
Ectopic Gestation, and Pelvic Hematocele . . . . . . . . . . . . . . . 462
Education Law, The New Medical . . 761
Electricity in Gleet and Impotence . . 33
Electricity in the Diseases of Women, 544
Electricity in Medicine and Surgery, Remarks on the Use of . . . . . . 288

PAGE.

Empyema, Operations for, particularly their After-Treatment, Translation . 513
Endometritis, Value of the Diagnostic Tampon in the Recognition of Chronic . . . . . . . . . . . . . 649
Ethics of Medical Visiting, The . . . . . . . . . . . . . . 264, 337, 419
Excessive Venery, Masturbation and Continence . . . . . . . . . . . . 175
Excretion and Absorption of Antipyrin . . . . . . . . . . . . . . . 43

F.

Favorite Prescriptions of Distinguished Practitioners, etc. . . . . . . . . . 490
Female Sexual Organs, Diseases of the Skin, Associated with . . . . . . 380
Fetid Sweating of the Feet . . . . . 35
Forceps Delivery . . . . . . . . . . 507
Is the Frequent Use of, Abusive? 340
Frederick, Case of the Late Emperor 408

G.

Galvanic Current in the Human Organism, Diffusion of . . . . . . . . . 541
Giddiness . . . . . . . . . . . 488, 621
Gleet, Electricity in, etc. . . . . . . 33
Goitre, Intraglandular Extirpation of, Socin's Method . . . . . . . . . 252
Gonorrheal Peritonitis . . . . . . . 212
Infection in Women . . . . . . 488
Greene, W. D., M. D, Forceps Delivery . . . . . . . . . . . . 507
Gynecic Element in Psychiatry, with Suggestions for Asylum Reform . . 569
Gynecology, A System of, by American Authors . . . . . . . . . . . . . 422

H.

Hand-Book of the Diagnosis and Treatment of Diseases of the Throat, Nose, and Naso-Pharynx . . . . . 482
Hartwig, Marcell, M. D., Management of Inflammatory Troubles in the Uterine Appendages . . . . . . . 234
Harrington, F. A., M. D., Dangers of Antipyrin, Translation . . . . . . 26
Hubbell, Alvin A., M. D., A Blow on the Ear, with Peculiar Reflex Symptoms . . . . . . . . . . . . . . . 202
Humerus, Epiphyseal Fracture of, etc. 431
Hurd, Arthur W., M. D., Malarial Paralysis . . . . . . . . . . . . . 19
Hydrocephalus, Dystocia, Due to Male Type of Pelvis, and . . . . . . . 454
Hymen, Alarming Hemorrhage from Rupture of the, during First Coitus, 250
Hysterectomy for Carcinoma Uteri, A Successful Vaginal . . . . . . . . 283

PAGE.

### I.

Ichthyol: Its Internal and External Uses . . . . . . . . . . . . . . 84
Impotence, Electricity in . . . . . . 33
Incontinence of Urine, A New Method of Treating, in Women . . . . . . 34
Indictment and the Defense . . . . . 219
India Fever, "Boo-Khar" . . . . . 665
Inflammatory Troubles in the Uterine Appendages, Management of . . . 234
Ingraham, Henry D., M. D., Introductory Lecture delivered at the Medical Department of Niagara University, Session of 1888 . . . . 107
Ingluvin in the Vomiting of Pregnancy, 542
Inoculation—Vaccination . . . . . . 581
Insomnia, Sulfonal in . . . . . . . . 210
Instruments—Allen Surgical Pump . . 121
Aspirator etc., . . . . . 428
Speculum, A Self-retaining Nasal . . . . . . 543
Syringe Point, The Star Recurrent Vaginal . . 543
Mechanical Nasal Saw . 671
International Medical Annual and Practitioner's Index . . . . . . . 629
—Pocket Medical Formulary . . 565
Interpreter, The Physician's . . . . . 178
Intestinal Disinfectant, Calomel as an 33
Intraglandular Extirpation of Goitre, Socin's Method, Translation . . . . 252

### J.

Jacobson, Nathan, M. D., Tracheotomy in Croup . . . . . . . . . . . . 1
Jenks Prize Essay . . . . . . . . . 680
Johns Hopkins Hospital . . . . . . 707

### K.

Keratitis Contagiosa in Cattle . . . 499
Kidney, Diseases of . . . . . . . . 493
Kirk's Hand-Book of Physiology . . 628
Krauss, Wm. C., M. D., A New Procedure with the Paraffine Method, 203
Tubercular Nodules of the Cerebellum . . . . . . . . . . . . 439
The Peculiarities of Chronic Cocaine Poisoning . . . . . . . 527
Treatment of Locomotor Ataxia by Suspension . . . . . . . . . 669

### L.

Laparatomy, etc., Translation . . . . 308
Locomotor Ataxia by Suspension, Treatment of . . . . . . . . . . 669
Leçons de Gynecologie Operatoire . . 491
Lecture, Introductory, delivered at the Medical Department, Niagara University, Session of 1888 . . . . . . 107
Life Insurance Examiner, etc. . . . . 359
Liver, Diseases of . . . . . . . . . 179

PAGE.

### M.

Malarial Paralysis . . . . . . . . . . 19
Manual of Dietetics for Physicians, Mothers and Nurses . . . . . . . 360
Manual of Instruction in the Principles of Prompt Aid to the Injured . 627
Materia Medica, Pharmacy, etc. . . . 495
Medical Microscopists . . . . . . . 259
Diagnosis . . . . . . . . . . . 358
Medical and Surgical History of the War of the Rebellion . . . . . . . 93
Medical Society of the State of New York . . . . . . . . . . . . . 474
Medical Colleges . . 85, 88, 170, 217, 220
Medical Education Law, The New . 761
Medical College Notes . . . . . . . 763
Medical Press of Western New York, Combination with . . . . . . . . 759
Meeting of the American Medical Association, The Newport . . . . . 760
Merck's Index of Fine Chemicals and Drugs . . . . . . . . . . . . . 630
Microscope, Important Discovery by the . . . . . . . . . . . . . . . 165
Milk, Feeding Infants with Asses' . . 537
Miller, John A., M. Sc., Ph. D., Antipyrin and Sweet Spirits of Nitre . 453
Miller, John A., M. D., Original Lecture on Ptomaines . . . . . . . 753
Monencephalic Thoradelphus . . . . 32
Moore, Edward M., M. D., Surgery of the Upper Extremities, 363, 431, 504
Mynter, Herman, M. D., Review of Surgical Cases Treated in the Buffalo General Hospital from March to July, 1888 . . . . . . . . . . . 49
Mynter, Herman, M. D., Reduction en masse . . . . . . . . . . . . . . 245
Mynter, Herman, M. D., Operations for Empyema, particularly their After-treatment . . . . . . . . . 513
Myrtol . . . . . . . . . . . . . . 542
Myxedema . . . . . . . . . . . . . 215
Myths and Modern Therapeutics . . . 62

### N.

Names that Mislead . . . . . . . . 211
National Formulary of Unofficinal Preparations . . . . . . . . . . . 95
Nervous Diseases, Lectures on, from the Standpoint of Cerebral and Spinal Localization, etc. . . . . . . . . 697
Nervous Exhaustion, Neurasthenia . . 295
A Practical Treatise on . . . . . 490
Neuralgia, Treatise on Headache and . 490
Neurasthenia, or Nervous Exhaustion . 295
Newport Meeting, The, of the American Medical Association . . . . . 760
New Medical Education Law, The . . 761
Nitre, Antipyrin and Sweet Spirits of 453

PAGE.

Nose, Foreign Bodies in . . . . . . 542
Noses, The Cure of Crooked and Deformed . . . . . . . . . . . 713

### O.

Obstetrics, Theory and Practice of, etc. 562
A System of . . . . . . . . . 45
Ophthalmologists in Paris . . . . . . 214
Ophthalmoscope, Theory and Practice of the . . . . . . . . . . . . . . . 179
Ovariotomy Work, Notes on Three Years of, in the Samaritan Free Hospital: 82 cases without a death . 81
—and Marriage . . . . . . . . . 163

### P.

Palpation of the Uterus made Easier by Extension of the Rectum . . . . 213
Paralysis, Malarial . . . . . . . . . . 19
Paraffine Method, A New Procedure with the . . . . . . . . . . . . . 203
Pathological Anatomy of Diphtheritic Paralysis, On the, Translation . . . 317
Patho-Biological Laboratory, Nebraska, Reports of . . . . . . . . 224
Pedigree of Disease . . . . . . . . . 356
Pelvic Abscess, A Contribution to the Study of . . . . . . . . . . . . 369
Peritomy . . . . . . . . . . . . . . 214
Perityphlitic Abscess, Case of . . . . 525
Physics and Chemistry, Essentials of . 630
Physicians' Pocket Day-Book and Ledger Combined . . . . . . 279, 358
Physiology, Text-Book of Human . . 176
Phthisiology, Hand-Book of Historical and Geographical . . . . . . . . . 274
Poisoning, The Peculiarities of Chronic Cocaine . . . . . . . . . . . . . . 527
Poisons, Druggists', and the Prescription of . . . . . . . . . . . . . . 212
Potter, Frank Hamilton, M. D., Report of a Case of Congenital Bony Occlusion of the Anterior Nares, 74
Notes on the Treatment of Acute Coryza . . . . . . . . . . . . 302
Mechanical Nasal Saw . . . . . . 671
A Self-Retaining Nasal Speculum, 543
Potter, Wm. Warren, M. D., . . . . 121
Preferred Creditors . . . . . . . . . . 758
Proper Use of Antiseptics in Puerperal Diseases, What is the . . . . . . 181
Ptomaines, by John A. Miller, M. D. . 753
Price, Joseph, M. D., A Consideration of Some of the Recent Work in Abdominal Surgery . . . . . . . . . 229
Price, Mordecai, M. D., Amputation of Thigh and Leg . . . . . . . . . 457
Prize, The Wm. F. Jenks . . . . . . 497
Proposed National Patho-Biological Laboratory . . . . . . . . . . . . 261
Psychiatry, The Gynecic Element in . 569

PAGE.

Psychic Life of Microörganism . . . 629
Pulmonary Tuberculosis, The Principles of Treatment in, with Some Observation on its Etiology . . . . 633
Purified Water . . . . . . . . . . . 461

### Q.

Quinine, Use of Antipyrin with . . . 461
Quiz Compends . . . . . . . . . . . 279

### R.

Ranney, Ambrose L., M. D., Letter from . . . . . . . . . . . . . . . 757
Reduction en masse . . . . . . . . . 245
Rectal Injection, Collapse from . . . 456
Reed, Chas. A. L., M. D., The Gynecic Element in Psychiatry . . . . . . 569
Reference Hand-Book of the Medical Sciences . . . . . . . . . . . . 48, 563
Regimen of the Japanese Soldiers . . 164
Reviewer Reviewed . . . . . . 611, 673
Rhinitis, Membranous . . . . . . . 538
Rigidity of the Os, Spasmodic . . . . 212
Roberts, John B., M. D., The Cure of Crooked and otherwise Deformed Noses . . . . . . . . . . . . . . 713
Rohé, George H., M. D., Disorders of the Skin associated with Diseases of the Female Sexual Organs . . . . 380
*Rupture* of the Hymen during First Coitus, Alarming Hemorrhage from 250
—of the Uterus, Report of Case, Laparatomy, Recovery . . . . . 308

### S.

Samaritan Free Hospital: Notes on Three Years' Ovariotomy Work in the . . . . . . . . . . . . . . 81
Scapula, Fractures of . . . . . . . . 504
Schultze, Dr., B. S., The Diagnostic Tampon; Its Value in the Recognition of Chronic Endometritis . . . 649
Scoliosis, Translation . . . . . . . . 252
Skin, Diseases of, associated with Disorders of the Female Sexual Organs 380
Skin Diseases, Photographic Illustrations of . . . . . . . . . 46, 278, 703
Common Diseases of . . . . . . 356
Hand-Book of the Diagnosis and Treatment of . . . . . . . . 631
Small-Pox in Buffalo . . . . . 40, 86, 97
Smith, Eugene, A., M. D., Dystocia, due to Male Type of Pelvis and Hydrocephalus . . . . . . . . . . 454
Smith, Eugene A., M. D., A Case of Perityphlitic Abscess . . . . . . . 525
Snow, Irving M., M. D., Chorea . . . 187
Socin's Method, Intraglandular Extirpation of Goitre of, Translation . . 252
Sozoiodol, a Substitute for Iodoform . 213

PAGE.
Speculum, A Self-Retaining Nasal . . 543
Sponges, Substitute for . . . . . . . 35
Sprains, Treatise on . . . . . . . . . 701
Stockton, Charles G., M. D., On the Nature of Croupous Inflammation with reference to the Puerperium . . 749
Street-cars a Menace to Public Health, 477
Successful Vaginal Hysterectomy for Carcinoma Uteri . . . . . . . . . . 283
Suggestive Therapeutics, Hypnotism . 623
Sulfonal in Insomnia . . . . . . . . 210
Surgical Cases Treated in the Buffalo General Hospital from March to July, 1888 . . . . . . . . . . . . . . 49
Surgery of the Upper Extremities, Bone, Part I., Treatment of the Clavicle when Fractured or Dislocated . 363
Part II., Epiphyseal Fracture of the Superior Extremity of the Humerus . . . . . . . . . . 431
Part III., Fracture of the Scapula 504
Surgery, The Operations of . . . . . 626
Sweating of the Feet, Fetid . . . . . 53
Syphilis by Conception, Translation . . 517
Syphilis, Infection of Physicians with 616
Syringe Point, Star Recurrent Vaginal 543

**T.**

Tait, Mr. Lawson, Correspondence . . 337
Tampon, The Diagnostic, Its Value in the Recognition of—Chronic Endometritis . . . . . . . . . . . 649
Therapeutics, Hand-Book of . . . . . 625
Therapeutics, Myths and Modern . . 62
Thrombus, The White, a Novel Primary Affection . . . . . . . . . 460
Tracheotomy in Croup . . . . . . . 1
Traumatic Tetanus, Case of, with Recovery . . . . . . . . . . . . . 199
Transactions of the Medical Society of the State of New York, 1888 . . . 94
Transactions of the Medical Association of Missouri, 1888 . . . . . . 225
Transactions of the Medical and Chirurgical Faculty of Maryland, 1888, 225
Transactions of the Medical Association of American Physicians and Surgeons, 1888 . . . . . . . . . 492
Tremaine, W. S., M. D., Remarks on the Use of Electricity in Medicine and Surgery, . . . . . . . . . . 288
Tobie, Edward, M. D., Obituary . . . 693
Tubercular Nodules of the Cerebellum, 439
Tubercular Patients . . . . . . . . 32
Tuberculosis, The Principles of Treatment in Pulmonary, with Some Observations on Its Etiology . . . . 633

**U.**

University of Buffalo, Medical Department . . . . . . . . . . . . . . . 556
University of Niagara, Medical Department . . . . . . . . . . . . 558, 614

PAGE.
Urine, A New Color-reaction of the 460
Uterus, Palpation of the, made Easier by Extension of the Rectum . . . . 213
Uterine Appendages, Management of Inflammatory Troubles in the . . . 234
Alkaloids in . . . . . . . . . . 32
Uterus, Remarks on Rupture of, with Report of a Case, Translation . . . 308
Inversion of . . . . . . . . . . . 610
Uterus, Pathology and Treatment of Displacements of the . . . . . . . 486

**V.**

Vaccination, Some Remarks on . . . 97
Compulsory in China . . . . . . 212
Inoculation . . . . . . . . . . . 581
Vaginal Hysterectomy for Carcinoma Uteri, A Successful . . . . . . . . 283
Vest-Pocket Anatomist . . . . . . . 359
Visiting List . . . . . . . . . 280, 427
Volume XXVIII. . . . . . . . . . . 35
Volume XXVIII., The Close of . . . 759

**W.**

Water, The Relations of, in the Bodily Economy . . . . . . . . . . . . . 539
Wathen, Wm. H., A Successful Vaginal Hysterectomy for Carcinoma Uteri . . . . . . . . . . . . . . 283
Why He was so Lean, . . . . . . . . 253
Women, A New Method of Treating Incontinence of Urine in . . . . . 34
Treatise on the Diseases of . . 345

### Editorials :

American Association of Obstetricians and Gynecologists . . . . . . . . . 268
Campbell, Dr. Frederick R., In Memoriam, 166; Successor to the Late . 172
Cesarean Section, The Abuse of . . . 262
Charter Revision Commission and the Buffalo Medical and Surgical Association . . . . . . . . . . . . . . 479
Chemist, An Expert . . . . . . . . 171
Cocaine—what to Use . . . . . . . 88
Colleges, Medical —The Winter Session of Lectures, 85; Opening of the Annual Sessions, 170; The Extended Course of Study in, 217; New York Post-Graduate, 88; Dental Department Meharry, 220; Cincinnati Polyclinic . . . . . . . . . . 617
Congress of American Physicians and Surgeons . . . . . . . . . . . 37, 169
Davidson, Dr., Library of . . . . 41, 89
Ectopic Gestation, and Pelvic Hematocele, etc. . . . . . . . . . . . . 463
Electricity in the Diseases of Women . 544
Ethics of Medical Visiting . . . 264, 419
Ewing, Dr. A. M., Course of Lectures 343
Forceps, Is the Frequent Use of, Abusive? . . . . . . . . . . . . 340

PAGE.

Frederick, Case of the Late Emperor . . . . . . . . . . . . 219, 408
Indictment and the Defense . . . . . 219
Jenks Prize Essay . . . . . . . . . . 680
Miller, Prof. John A. . . . . . . . . 269
Myxedema . . . . . . . . . . . . 215
Patho-Biological Laboratory, The Proposed National . . . . . . . . . . 260
Physicians' Protective Alliance . . . 267
Reviewer Reviewed—" J. C. R." and Tait's Ectopic Pregnancy . . . 611, 673
Roe, Dr. J. O. . . . . . . . . . . . 220
Roosa, Dr. D. B. St. John . . . . . 267
Small-Pox in Buffalo . . . . . . . 40, 86
Society, The Medical, of the State of New York . . . . . . . . . . . . 474
Street-cars a Menace to Public Health . . . . . . . . . . . . . . . 477, 617
Syphilis, The Infection of Physicians with . . . . . . . . . . . . . . . 616
Tobie, Edward, M. D., In Memoriam . 693
University of Buffalo, Medical Department, Alumni Association, Commencement . . . . . . . . . . . . 556
Medical Dep't of Niagara . 614, 558

### Societies :

Academy of Medicine, Paris . . 208, 535
American Medical Association . . . . . . . . . . . . . . . . . 273, 560, 668
American Medical Editors' Association. 695
American Association of Obstetricians and Gynecologists . . . . . . . 79, 268
American Association of Obstetricians and Gynecologists, Proceedings of . 125
American Association of Obstetricians and Gynecologists, Transactions of . 632
American Public Health Association . . . . . . . . . . . . . 223, 705
American Academy of Medicine . . . 224
American Pharmaceutical Association . 91
American Rhinological Association . . 91
American Society of Microscopists . . 618
Alumni Association, Medical Department, Niagara University . . . 559–560
Alumni Association, Medical Department, University of Buffalo . . . . 556
British Laryngological and Rhinological Association . . . . . . . . . 43
Buffalo Obstetrical Society, Report of . 393
Buffalo Pathological and Clinical Society. . . . . . . . . . . . . . . . 173
Buffalo Medical and Surgical Association. . . . 272, 344, 407, 479, 560, 618
Buffalo Medical and Surgical Association, Proceedings of . . . . . . . . . . . . . 77, 123, 206, 253, 396 648
Buffalo Medical and Surgical Association, Programme of . . . . . . . 174
Buffalo Medical and Surgical Association . . . . . . . . . . . . . . 763
Central New York Medical Association . . . . . . . . . . . . . . . 272

PAGE.

Central New York Medical Association, Proceedings of . . . . . . . . 321
Congress of American Physicians and Surgeons . . . . . . . . . . . . . 91
Congress of Otology and Laryngology . 618
Erie County Medical Society. [See Medical Society of.]
International Congress of Therapeutics and Materia Medica . . . . . . . . 617
International Congress of Hydrology and Climatology . . . . . . . . . 561
New York State Medical Society, Report of . . . . . . . . . . . . . . 474
New York State Medical Society, Programme of . . . . . . . . . . 405, 344
New York Polyclinic and Hospital . . 175
Niagara University, Medical Department . . . . . . . . . . . . 558, 614
Medical Society of Erie County . . . 344
Medical Society of Erie County, Proceedings of . . . . . . . . . . 400, 672
Medical Society of Pennsylvania, State 618
Medical Society of Michigan, State . 619
Medical Society of Nebraska, State . . 619
Medical Society of Ohio, State . . . . 619
Mississippi Valley Medical Association 91
Ontario Medical Association . . . . . 619
Philadelphia County Medical Society, Report of . . . . . . . . . . . . . 329
Philadelphia Obstetrical Society, Report of . . . . . . . . . . . 528, 604
Southern Surgical and Gynecological Association . . . , . 91, 175, 272, 344

### Obituary Record:

Campbell, Dr. F. R. . . . . . . . . . 166
Dalton, Dr. John C. . . . . . . . . . 481
Fothergill, Dr. J. Milner . . . . . . 42
Garnett, Dr. A. Y. P. . . . . . . . . 43
Gross, Dr. Samuel Weissel . . . . . 620
Jewett, Dr. Harvey . . . . . . . . 223
Langworthy, Dr. Henry H. . . . . . 561
Lambert, Mr. Jordan W. . . . . . . 481
Lynch, Dr. John S. . . . . . . . . 223
Matthews, Mr. James N . . . . . . 344
Sands, Dr. Henry B. . . . . . . . . 273
Southworth, Dr. John W. . . . . . . 561
Tobie, Dr. Edward . . . . . . . . 693
Williams, Dr. Elkanah . . . . . . . 223

### Personals :

Andrews, Dr. J. B. . . . . . . . . . 694
Allen, Dr. Cyrus A. . . . . . . . . 697
Biggs, Dr. Hermann M. . . . . 481, 619
Carstens, Dr. J. Henry . . . . . . . 221
Clark, Dr. Edward . . . . . . . . 272
Clark, Dr A. M. . . . . . . . . . 695
Davis, Dr. N. S. . . . . . . . . . . 271
Ewing, Dr. A. M. . . . . . . . . . 343
Falk, Dr. J. . . . . . . . . . . . 697
Flagg, Dr. J. D. . . . . . . . . . . 697
Frederick, Dr. C. C. . . . . . . . 780

PAGE.

Greene, Dr. J. C. . . . . . . . . . . 90
Greene, Dr. S. S. . . . . . . . . . . 90
Gumaer, Dr. A. G. . . . . . . . . . 90
Hammond, Dr. Wm. A. . . . . 174, 421
Hare, Dr. Hobart A. . . . . . . . . 780
Hasslock, Mr. Herman A. . . . . . . 343
Hovey, Dr. L. B. . . . . . . . . . . 90
Howe, Dr. Lucien . . . . . . . . . 173
Horsley, Mr. Victor . . . . . . . . 222
Hubbell, Dr. A. A. . . . . . . . . . 695
Krauss, Dr. William C. . . . . . . . 695
Lautz, Mr. F. C. M. . . . . . . . . 221
Loomis, Dr. H. P. . . . . . . . . . 619
Lusk, Dr. Wm. T. . . . . . . . . . 221
Matthews, Dr. J. M. . . . . . . . . 694
Miller, Dr. J. A. . . . . . . . . . 269
Mitchell, Dr. S. Weir . . . . . . . 421
Morrow, Dr. Prince A. . . . . . . . 620
Mynter, Dr. Herman . . . . . . . . 481
McMurtry, Dr. L. S. . . . . . . 174, 619
O'Dwyer, Dr. Joseph . . . . . . . . 271
Osler, Dr. Wm. . . . . . . . . . . 222
Opie, Dr. Thomas . . . . . . . . . 221
Parker, Dr. Wm. A. . . . . . . . . 562
Parmenter, Dr. John . . . . . . . . 480
Park, Dr. Roswell . . . . . . . 620, 694
Peck, Dr. George S. . . . . . . . . 695
Persons, Dr. A. E. . . . . . . . . . 695
Prudden, Dr. T. M. . . . . . . . . 619
Price, Dr. Joseph . . . . . . . . . 222
Putnam, Dr. James W. . . . . . . . 480
Reed, Charles A. L. . . . . . . 271, 481
Ring, Dr. Charles A. . . . . . . . . 562
Roe, Dr. J. O. . . . . . . . . . . 220
Rogers, Dr. Caroline S. . . . . . . . 90
Sheridan, General P. H. . . . . . . 42
Sutton, Dr. R. Stansbury . . . . . . 694
Tait, Mr. Lawson . . . . . . . . . 42
Thomas, Dr. T. Gaillard . . . . . . 272
Wathen, Dr. Wm. H. . . . . . . . . 272
Wende, Dr. Ernest . . . . . . . . . 271
Wheeler, Dr. William A. . . . . . . 762
Wiegel, Dr. Louis A. . . . . . . . . 343

### Reviews:

Annual Reports of State Boards of Health; Pennsylvania, Second; Michigan, Fifteenth; New York, Eighth; 1888 . . . . . . . . . . . 277
Baker [and Harris]: Kirk's Hand-Book of Physiology . . . . . . 628
Beard: A Practical Treatise on Nervous Exhaustion . . . . . . . . . 490
Bedside Record . . . . . . . . . . 95
Bernheim [Herter, Translation,]: Suggestive Therapeutics. Hypnotism . 623
Billings: Reports from the Patho-Biological Laboratory, University of Nebraska . . . . . . . . . . . . 224
Billington, C. E.: Diphtheria—Its Nature and Treatment . . . . . . 767
Binet [McCormack, Translation,]: The Psychic Life of Microörganism, . . 629
Bowen: Hand-Book of Materia Medica, Pharmacy and Therapeutics . . . . . . . . . . . . . . . 495
Brown: Medical Diagnosis—A Manual of Clinical Methods . . . . 358
Buck: Reference Hand-Book of the Medical Sciences, Vol. VI. . . . . . 48
Buck: Reference Hand-Book of the Medical Sciences, Vol. VII. . . . 563
Buck, Albert H.: A Manual of Diseases of the Ear . . . . . . . . . 772
Burnett: Diseases and Injuries of the Ear; their Prevention and Cure . . 704
Burt: Exploration of the Chest in Health and Disease . . . . . . . 564
Cazeaux [and Tarnier]: The Theory and Practice of Obstetrics, etc. . . 562
Charcot [Hurd, Translation,]: Clinical Lectures on Certain Diseases of the Nervous System . . . . . . . . . 276
Claiborne: The Theory and Practice of the Ophthalmoscope . . . . . . 179
Corning: A Treatise on Headache and Neuralgia . . . . . . . . . 490
Cutler: Essentials of Physics and Chemistry . . . . . . . . . . 630
Disinfection and Disinfectants . . . . 226
Diet Tables . . . . . . . . . . . 227
Doty: A Manual of Instruction in the Principles of Prompt Aid to the Injured . . . . . . . . . . . . 627
Dujardin-Beaumetz [Hurd, Translation]: Diseases of the Liver . . . 179
Modern Treatment of Diseases of the Kidney . . . . . . . . . 493
Evans: Hand-Book of Historical and and Geographical Phthisiology . . . 274
Favorite Prescriptions of Distinguished Practitioners, with Notes, etc. . . . 490
Ferrand: Varieties and Treatment of Bronchitis . . . . . . . . . . . . 356
Flint: A Text-Book of Human Physiology . . . . . . . . . . . . 176
Foster: An Illustrated Encyclopedic Medical Dictionary . . . . . . . 43
Fox: Photographic Illustrations of Skin Diseases, etc. . . . . . . . . 278
Fox: Quiz Compends . . . . . . . 279
Gould: Quiz Compends . . . . . . 279
Harris [and Baker]: Kirk's Hand-Book of Physiology . . . . . . . 628
Hirst: A System of Obstetrics . . . . 45
Howe: Excessive Venery, Masturbation, and Continence . . . . . . . 175
Hutchinson: The Pedigree of Disease 356
Ideals of the Republic . . . . . . . 777
International Medical Annual and Practitioners' Index . . . . . . . 629
Jacobson: The Operations of Surgery, etc. . . . . . . . . . . . . . . 626
Jaenton: Albuminuria in Bright's Disease . . . . . . . . . . . . 488, 621
James: American Resorts, with Notes upon their Climate . . . . . . . 702

PAGE.
Kilmer: Physicians' Pocket Day-Book and Ledger Combined . . . 358
Leidy, Joseph: An Elementary Treatise of Human Anatomy . . . . . 775
Leonard: The Vest-Pocket Anatomist 359
Leonard: The Physicians' Pocket Day-Book . . . . . . . . . . . 279
Loomis: Bright's Disease . . . . . . 493
Mann: A System of Gynecology by American Authors . . . . . . . . . 422
Marcy, Henry O.: A Treatise on Hernia . . . . . . . . . . . . . 764
Merck's Index of Fine Chemicals and Drugs, etc. . . . . . . . . . . . . . 630
Morrow: Atlas of Venereal and Skin Diseases . . . . . . . . 46, 278, 703
Morrow, Prince A.: Atlas of Venereal and Skin Diseases . . . . . . . . 776
Moullin: Treatise on Sprains . . . . 701
Mundé: The Theory and Practice of Obstetrics [Cazeaux and Tarnier] . 562
National Formulary of Unofficinal Preparations . . . . . . . . . . . 95
Palmer: Favorite Prescriptions of Distinguished Practitioners, etc. . . 490
Pritchard: Manual of Dietetics for Physicians, Mothers, and Nurses . . 360
Raue, C. G.: Psychology as a Natural Science Applied to the Solution of Occult Phenomena . . . . . . . . 774
Ranney: The Applied Anatomy of the the Nervous System . . . . . . . 48
Ranney: Lectures on Nervous Diseases from the Standpoint of Cerebral, etc. . . . . . . . . . . . . . 697
Ringer: A Hand-Book of Therapeu tics . . . . . . . . . . . . . . . . 625
Rockwell, A. D.: A Practical Treatise on Nervous Exhaustion (Neurasthenia) . . . . . . . . . . . . . . 490
Sajous: Annual of the Universal Medical Sciences . . . . . . . . . . 92
Schultze: The Pathology and Treatment of Displacements of the Uterus . . . . . . . . . . . . . . 486
Seiler: Hand-Book of the Diagnosis and Treatment of Diseases of the Throat, Nose, and Naso-Pharynx . . 482
Sexton: The Ear and its Diseases . . 275
Simon . Common Diseases of the Skin. 356
Sinclair: Gonorrheal Infection in Women . . . . . . . . . . . . . 488
Skene: Treatise on the Diseases of Women . . . . . . . . . . . . . 345
Stewart: On Giddiness . . . . . 488, 621
Stewart: Clinical Lectures on Albuminuria . . . . . . . . . . . . . 494, 621
Smart: Medical and Surgical History of the War of the Rebellion . . . 93
Stillman: Life Insurance Examiner—A Practical Treatise, etc. . . . . . 359
Tarnier: The Theory and Practice of Obstetrics, etc. . . . . . . . . . 582
Thompson: Preventive Treatment of Calculous Disease, and the Use of Solvent Remedies . . . . . . . . 701

PAGE.
Tiemann: The American Armamentarium Chirurgicum . . . . . . . . 699
Transactions of the Medical Society of the State of New York, 1888 . . 94
Transactions of the Medical Association of Missouri, 1888 . . . . . . 225
Transactions of the Medical and Chirurgical Faculty of Maryland, 1888 . . . . . . . . . . . . . . . 225
Transactions of the Association of American Physicians, 1888 . . . . 492
Transactions American Association Obstetricians and Gynecologists. Volume I. 1888 . . . . . . . . . 770
Van Harlingen: Hand-Book of the Diagnosis and Treatment of Skin Diseases . . . . . . . . . . . . . 631
Visiting List [Lindsay & Blakiston's], 280
Visiting List and Call Record [Davis], 427
Vol. V. The Physician's Interpreter, 179
Vulliet & Lutaud: Leçons de Gynecologie Operatoire . . . . . . . . 491
Witherstine: International Pocket Medical Formulary . . . . . . . 565
Wood: Therapeutics, etc. . . . . . 177
Ziemssen, H. Von: Pulmonary Tuberculosis . . . . . . . . . . . . . 777

### Journalistic and Literary:

Alabama Medical and Surgical Age . . 568
Analectic, The Medical . . . . . . . 342
Annals of Surgery . . . . . . . . . 708
Archives of Pediatrics . . . . . . . 632
Archives of Gynecology . . . . . . 269
Atlanta Medical and Surgical Journal . 568
Canada Medical Record . . . . . . 270
Canadian Practitioner . . . . . 343, 632
Catalogue of Medical Books and Journals . . . . . . . . . . . . . . . 496
Chicago Law Times . . . . . . . . 568
Cosmopolitan . . . . . . . . . 632, 706
Cutaneous and Genito-Urinary Diseases, The Journal of . . . . . . . 428
Cyclopedia of the Diseases of Children, 428
Dietetic Gazette . . . . . . . . . . 696
Journal of the Respiratory Organs . . 568
Kansas Medical Journal . . . . . . 696
Maritime Medical News . . . . . . 270
Medical Register . . . . . . . . . 696
Medical Age . . . . . . . . . . . . 568
Montreal Medical Journal . . . . . . 173
New York Medical Record . . . . . 173
Open Court . . . . . . . . . . 495, 496
Pacific Medical Journal . . . . . . 568
Philadelphia Medical Times . . . . . 696
Register of the United States, Medical and Surgical . . . . . . . . . . 631
St. Louis Polyclinic . . . . . . . . 696
St. Louis Courier of Medicine . . . . 220
Southern California Practitioner . . . 270
The American Journal of Obstetrics . 780
The Cosmopolitan . . . . . . . . . 780
The Medical News . . . . . . . . 780
The Times and Register . . . . . . 780
Times and Register . . . . . . . . 696
University Medical Magazine . . . . 221

The thirty-seventh meeting of the American Association for the Advancement of Science will be held at Cleveland, beginning Wednesday, August 15th, and closing Tuesday evening, August 21, 1888.

---

